# Essential services provided and costs of facility-based maternity waiting homes in Ethiopia

**DOI:** 10.11604/pamj.2021.39.109.22851

**Published:** 2021-06-07

**Authors:** Biniam Getachew, Tippawan Liabsuetrakul

**Affiliations:** 1 Epidemiology Unit, Faculty of Medicine, Prince of Songkla University, Hat Yai, Songkhla 90110, Thailand

**Keywords:** Cost, essential services, maternity waiting home

## Abstract

**Introduction:**

the objective was to describe establishment cost, essential services provided and operating costs of maternity waiting homes (MWH) in Ethiopia.

**Methods:**

a cross-sectional study was carried out from December 2017 to June 2018 in eight health facilities with maternity waiting homes (MWH) in the Gurage Zone of Ethiopia. MWH users exit interviews and observational checklists were used to collect data on essential services provided. Cost-related data were retrieved from relevant records in the health facilities.

**Results:**

most clinical services and basic amenities were available and provided for MWH users. The average capital costs of a MWH were $2,245 US with fixed costs of $1,476 US per year. The personnel cost for a MWH was $1,439 US per year. The average annual running cost of a MWH was $1,303 US per year. The average estimated MWH utilization and delivery costs was $16.9 US per woman.

**Conclusion:**

most MWHs provided essential clinical services and basic amenities. The majority of the cost of a MWH was attributed to building construction costs. If building cost is annualized, the unit cost of a MWH service is in an acceptable range which encourage government considering expansion of the service in rural area.

## Introduction

Maternal mortality remains a global issue and an important part of the Sustainable Development Goals [1]. Approximately 830 women die due to pregnancy and childbirth every day globally, of whom 99% are in developing countries [2]. The poor utilization of maternal health services in areas where maternal deaths are high is mainly due to barriers to access those services [3]. According to recent WHO recommendations to improve access to delivery care, many developing countries are instituting maternity waiting homes (MWH) [4]. The MWH is a separate lodging or accommodation within or nearby an existing health facility where women who are near to giving birth can await their labor [4, 5]. Establishment and running of MWHs is considered as a good strategy to overcome inaccessibility to childbirth centers due to distance, geography, seasonal barriers or the time of day, infrastructure, transport, financial barriers and communication barriers between referral points [4].

Typologies of the MWH and essential services provided vary within and across countries which leads to wide range of costs involved with providing MWH services [4, 5]. Most MWHs are established and run either by a local government, international donation and/or from support by the community [4, 6]. However, most studies on MWHs have not reported the overall costs to establish or run one of these facilities. One study from Lesotho reported that the average cost of constructing a MWH was $36,760 US, but the cost depended on whether a home was newly built or an existing structure was re-purposed. The initial operating cost of such a facility was then around $450 US for one MWH´s supplies and equipment [7]. Another report from Timor-Leste found the renovation cost to re-purpose an old building for MWH services ranged from $10,000 US to $15,000 US and $40,000 US to $50,000 US to build a new one [8].

Many years of experience, the availability of a large number of MWHs and having standard guideline of MWH implementation makes Ethiopia the right place to gather tangible evidence on the essential services provided, basic amenities, and establishment and operating costs of MWHs. Understanding the establishment and operating cost of MWHs will provide evidence for governments and other implementers for budgeting and planning. Importantly, such a study can provide lessons learned for countries with a similar context to Ethiopia where establishing MWHs would be very beneficial to pregnant women. Hence, the aim of this study was to describe essential services provided and examine the establishment and operating costs of MWHs in Ethiopia.

## Methods

### Study design, setting and period

A cross-sectional study was carried out in eight health facilities with a MWH in Ethiopia, namely: Attat Our Lady of Lourdes Catholic Primary Hospital, Gunchere Primary Hospital, Emdibir Health Center, Agena Health Center, Butajira General Hospital, Bui Primary Hospital, Kela Health Center and Koshe Health Center. The study setting is located in the Gurage Zone of the Southern Nations, Nationalities, and People´s Region, Ethiopia. The study period was from December 2017 to June 2018. The hospitals provide comprehensive emergency obstetric and newborn care, while the health centers provide basic emergency and newborn care. Since all health facilities are located in rural areas, there are limited resources in all study settings.

### Sample size

The sample size was calculated using the one group mean formula for the cost of MWH utilization with an estimate of standard deviation of 50 ETB (equivalent to $2 US) of individual cost of MWH utilization and 20% estimation of true mean difference, a design effect of 1.5 and a 10% non-response rate, giving a result of 40 women at each health facility being required. Due to a higher number of MWH users during the study period in Attat hospital (5 times more) and Butajira hospital (3 times more), data were collected from 560 women from 8 health facilities, 200 women from Attat hospital, 120 women from Butajira hospital and 40 women each from the other 6 health facilities. All postpartum women who stayed at the MWH then delivered at the associated labor ward during the study period were included in this study, except those who were unable to communicate due to any health-related or personal problems.

### Variables

The list of essential services provided was obtained from the Ethiopian Ministry of Health. The Ethiopia National Guideline for Establishment of Standardized MWHs includes the following essential services to be provided at a MWH: ANC, vital signs, iron/iron folate supplementation, health education, water, electricity and means of recreation such as television/radio and traditional coffee ceremony [9]. Standard MWHs are expected to have adequate air flow, windows allowing light, easy-to-clean floors, roofs and walls; have to accommodate at least one family member; and easily accessible clean and safe drinking water, toilets and bathrooms. Kitchens must be separate and with necessary cooking utensils [9].

The calculation of MWH building costs included the total cost estimation of the rooms used for the purpose of MWH which included a staying room, kitchen and toilet. The costs that were properly documented were retrieved from the health facilities´ administrative offices. For undocumented prices and community ‘in kind´ contributions, the costs were estimated by a construction expert with the help of information provided by the health facilities´ staffs. The annual cost of the buildings for the year 2018 were estimated for each of the eight MWHs considering a 3% discount rate since the year of construction [10]. Annualized cost of building construction was calculated to estimate the MWH annual cost, considering a MWH will serve for ten years. Due to lack of documentation for most equipment and supplies and furnishings and kitchen utensils, the price was estimated based on the current (2018) market price. Overhead costs included the cost of electricity and water expenses for one year. In most of the health facilities, there were no separate payment systems for overhead costs of MWHs, hence, an estimation was performed by considering the probable electricity and water consumption of MWH users. Annual personnel costs were calculated by using the gross salary and income of staff (basic salary, night shift differential and professional allowance) who work in the MWH and the percentage of time they spent working at the MWH. Running costs included the health facilities´ annual expenses for cleaning materials, toiletries, repairs and maintenance, provision of meals and other dispensable items. The costs of meal provision were estimated according to each MWH´s policy. In some MWHs, there was in kind support from the community which was converted into monetary value.

### Data collection

The sampled MWH users were invited to participate in the study and those who agreed signed a consent form. A structured questionnaire was used to interview the MWH users by trained research assistants and an observational checklist was used to gather information about available services in the MWHs. Cost-related data were collected using a structured questionnaire through document review and expert estimations. Every questionnaire was checked for completeness and consistency to ensure the good quality of the data.

### Data management and analysis

The data were entered in EpiData 3.1 using double entry and were analyzed using R version 3.5.1 (R Foundation for Statistical Computing, Vienna, Austria). The availability of clinical services and basic amenities in MWHs and characteristics of MWHs are presented in frequencies and percentages. The establishment and operating costs of MWH are presented in tables. To ensure the privacy of the health facilities, anonymous names in an alphabetic format were used. All costs were recorded in Ethiopian Birr (ETB) and later converted to United States dollars (USD) using an exchange rate of 1 ETB per 0.036 USD [11]. The overhead costs of MWHs and personnel costs were estimated for each month then converted to an annual figure. Considering all the mentioned costs above, the average costs of MWH service per year were estimated for the year 2018. Finally, the unit costs were estimated considering the monthly estimated cost of a MWH divided by the average number of MWH users per month. Average number of users obtained from the previous year report.

### Ethical considerations

Ethical approval was obtained from the Ethics Committee of the Faculty of Medicine, Prince of Songkla University, Thailand (EC no. 60-253-18-5). The MWH users were informed about the purpose of the study and their freedom and rights to decide whether they agreed to participate. Their data were kept anonymity and confidentiality.

### Funding

This work was partially supported by Graduate School Dissertation Funding, Prince of Songkla University. The funder had no role in this article.

## Results

### Essential services and basic amenities at MWHs

In this study, 560 MWH users were interviewed about the availability of essential services provided at MWHs and eight MWHs were observed for their provision of services. Of the 560 MWH users, their mean age was 27.51 years (±sd 5.14), 71.8% were housewives and 36.8% were illiterate. Table 1 shows the women´s utilization of available clinical services and basic amenities in MWHs. More than 90% of MWH users received ANC check-up and health education services while staying at the MWH. Almost all (97%) of the women reported the availability of water, electricity and toilets in the MWHs and rated them as being clean. Just over half of the women (56.1%) received either free or partially supported stays at their MWHs. Nearly two-fifths of the women reported that their food was cooked by MWH cleaners. Around 60% of the women indicated the availability of television/radio and traditional coffee ceremonies as a means of recreation in their MWHs.

**Table 1:** availability of clinical services and basic amenities in maternity waiting homes (MWHs)

Services	Number of MWH users (n=560)	Percentage
ANC check ups	544	97.1
Measure vital signs everyday	395	70.5
Provide iron/iron folate supplementation	424	75.7
Giving solutions for existing problems*	483	86.2
Provision of health education	526	93.9
Free/partially free food provided by the MWH	314	56.1
**Who cooks the meals at the MWH? (n=555)**		
MWH attendees	179	32.3
Families	148	26.6
MWH cleaners	228	41.1
Water provided	557	99.5
Electricity available	558	99.6
MWH is clean	551	98.4
**Who cleans the MWH**		
MWH attendees	90	16.1
Families	38	6.8
MWH cleaners	432	77.1
Toilet and bathroom are clean	544	97.1
**Who cleans the toilet and bathroom?**		
MWH attendees	33	5.9
Families	3	0.5
MWH cleaners	523	93.4
Healthcare providers	1	0.2
Television/radio provided	324	57.9
Traditional coffee ceremony available	348	62.1

* meals, family issues/quarrels, service complaints, etc.

### Characteristics of MWHs

Table 2 shows the characteristics and available amenities of the MWHs. All of the MWHs in this study were located inside pre-existing health facilities. One of the MWHs was a traditional hut, four of them were semi-modern and the remaining three were modern and well-furnished. Four of the MWHs had walls built from natural material (stone, mud, thatch and wood) and the other four were built from bricks. All of the MWHs were supported by the local communities, and had metal/corrugated iron roofs, allowing natural light during daytime, electricity, water, mattress and blankets, toilet and security guard. Adequate air flow, health teaching posters, a kitchen with cooking utensils and a place at least for one family member to stay together with the women were observed in the seven out of eight MWHs. A bathroom was available in 5 of the MWHs and only four MWHs had beds for the women while the remaining four had only a mattress on the floor.

**Table 2:** characteristics of maternity waiting homes (MWHs) (n = 8)

Characteristic	Number of MWHs
**Type of MWH**	
Traditional hut	1
Semi-modern	4
Modern and well-finished	3
**Walls of the MWH**	
Natural material (stone, mud, thatch, wood)	4
Concrete/bricks	4
Metal/corrugated iron roof	8
Allows natural light	8
Electricity available	8
Water supply	8
Mattress and blankets provided	8
Toilet	8
Security	8
Community support	8
Window(s) (adequate air flow)	7
Health teaching posters	7
Kitchen available	7
Kitchen has necessary cooking utensils	7
Space for at least one family member	7
Bathroom available	5
Bed provided	4

### Cost of MWHs

The costs for establishing and operating the MWHs are presented in Table 3. An overall estimate of building a MWH was $10,236 US before annualizing. The average building construction cost of a MWH was $1,024 US per year and the cost of furnishing and kitchen utensils was $1,221 US per year, giving an average capital costs of a MWH of $2,245US per year. The highest construction cost was $1,977 US and the lowest was $201 US. Average fixed costs for a MWH were $1,476 US per year with $38 US and $1,439 US per year for electricity/water and personnel costs, respectively. The average annual running cost of a MWH was $1,303 US, ranging from $427 to $2,265 US. The average annual cost of a MWH was $5,126 US (Table 3).

**Table 3:** establishment and operating costs of maternity waiting homes (MWHs)

Costs	Purpose	Hospitals				Health centers				
		A	B	C	D	E	F	G	H	Average
Capital cost (USD)	Building costs	1475	1977	1621	1945	368	267	201	335	1024±799
	Furnishing and kitchen utensils costs	1610	666	1574	1033	1095	1330	1414	1042	1221±320
Fixed/ overhead costs (USD)	Maintenance cost (10% of construction cost)	148	198	162	194	37	27	20	33	102±80
	Electricity and water costs per year	52	60	25	25	43	30	43	25	38±14
	Annual personnel cost	996	1272	3588	1500	876	1440	876	960	1439±903
Variable cost (USD)	Running cost	427	2265	427	2137	1282	1878	1581	427	1303±787
Total annual cost (USD)		4708	6438	7397	6834	3701	4971	4135	2822	5126± 1618

### Unit cost of MWHs

The average estimated MWH utilization and delivery costs were $16.9 US per woman. The highest unit costs were $28.5 US and lowest were $4.9 US per women per facility. This varying unit cost could be accredited to the number of MWH users, with higher numbers of MWH users resulting in lower per unit costs, and vice versa. Table 4 compares the actual unit costs and ideal (maximum capacity) unit costs if the health facilities accommodated the maximum number of MWH users per month.

**Table 4:** maternity waiting homes (MWH) service and its unit cost

Costs	Hospitals				Health centers				Average
	A	B	C	D	E	F	G	H	
Total monthly cost (USD)	392	537	616	570	308	414	345	235	427±134
Number of cases per month	80	30	25	20	40	18	17	21	31±21
Unit cost per case (USD)	4.9	17.9	24.7	28.5	17.1	10.3	20.3	11.2	16.9±7.8
Capacity of MWH user per month	80	40	50	40	40	30	25	30	-
Ideal unit cost per case (USD)	19	15	36	50	50	19	20	16	-

## Discussion

Basic clinical services and amenities were provided to most of MWH users during their stay. Not all MWHs provides meals to the users. The type of MWH varied between the sites, from traditional hut to semi-modern or modern and well furnished, either built from natural materials or concrete bricks. The average initial costs for construction and furnishing a MWH were $ 2,245 US. The annual operating cost of a MWH were $2,882 US. Basic clinical services for MWH services were available in most health facilities as recommended by the guidelines of WHO and Ministry of Health (Ethiopia) [9, 12] and most MWH users in our study reported having access to those services. A study from Ethiopia found that most MWHs had an electricity grid connection and toilet, however, less than half of them had water [13]. The similarity might be due to the MWHs sharing resources with the health facilities. A Zambian study reported that the majority of MWH users had access to water, electricity and a toilet [14], but as study from Malawi reported that MWH users could not access reliable water or electricity [15]. The provision of food at MWHs was heterogeneous. A study from Ethiopia reported that more than half of MWH users responded food was not affordable while staying at MWH [16] which was consistent with the findings from the studies in different countries found that the availability of food was the main reason women agreed or chose to use a MWH [15, 17-20] or a barrier for MWH utilization [6, 21].

MWHs have been implemented in many countries in all major continents including Africa, Asia, South America and North America [5]. However, the typology, services provided, availably of food and amenities at the MWHs varies within and among countries [4, 13]. In a few health facilities in Ethiopia and Zimbabwe, traditional style huts were built and provide the MWH service [5, 6], while other MWHs provide modern or semi-modern rooms with corrugated iron roofs [13, 22], One of the MWHs in our study was the traditional style hut while the other seven were semi-modern or modern buildings. Following the MWH national guideline of Ethiopia [9], all of the MWHs in our study setting were built inside a health facility.

There is a paucity of information on the cost to provide MWH facilities, and the few studies that have been done used different methods to calculate the cost so comparisons are difficult to make. In our study, we estimated the construction costs of the MWH regardless of sources of funding which can provide firsthand evidence for policymakers or stakeholders who plan to provide MWH services. In Lesotho, the average cost of constructing a MWH was $36,760 US [7], which was three times higher than our study. A report from Timor-Leste found the renovation of old buildings to re-purposed MWHs cost between $10-15,000 US, with new building costs as high as $50,000 US [8]. The different construction costs of building MWHs between those studies and our study were mainly due to the typology of the MWHs and the difference economic statuses of these countries. The fixed costs to run a MWH in Zambia was $543 US per year [23], whereas in our study the fixed costs were $1,477 US. The high dissimilarity of these fixed costs was at least partly due to the different ways of measuring the fixed costs.

The working hours of health professionals at the MWHs also vary among MWHs in different countries from full time to a small percent of their working time. In Nepal, the annual personnel cost was around $5,361 US (NOK 47,000) [24], whereas in our study it was only $1,439 US. This difference might be due to different salary levels for health professionals between the two countries. In one of our study settings, there were two full time midwifes working on shift which made the annual personnel costs of this facility much higher. In the rest of our MWHs, nurses/midwives were working at the MWH for 7-13% of their total working hours. In our study, the startup costs of the MWHs were found to be a huge additional cost for the health sector. However, after the establishment of a MWH, the operating costs per year was much smaller. Other studies reported that the establishment costs of MWHs were fully or partially supported by other stakeholders [6, 23].

The unit cost of MWH utilization and delivery service in our study was $16.9±7.8 US which was within the range of unit cost of vaginal delivery services reported in the synthesis of case studies from different sub-Saharan African countries, $2.7-33.9 US in Uganda, $10.2-24.0 US in Malawi, and $7.7-14.6 US in Ghana [25]. High cost in $57 US per delivery per health center was reported in another study from Ghana which could be explained by low services utilization [26]. The estimation of unit costs might vary depending on the number of MWH users during the study period and estimation of undocumented prices. During the study period, only two of the MWHs were accommodating women with their full capacity, which indicates underutilization of the MWH services in the other six MWHs. For the efficient utilization of the MWHs, provision of quality services and government policies encouraging women to use MWHs should be in place. As a basic supply and demand equilibrium principle, the higher the number of MWH users, the lower the unit cost [27]. Our study included both old and new MWHs could provide reliable estimates of the costs of MWHs. In addition, the study was conducted in rural areas which enabled it to estimate the construction and operating costs of MWHs in hard-to-reach areas where such services are most needed. Overall, this study provides important information for policymakers on the costs of establishing or expanding MWH services specifically for developing countries. The study had some limitations. First, there were a limitation in records of the actual costs of construction or renovation expenses of the MWHs that left us with no option other than professional estimations. In order to minimize error due to over or under estimations, we included construction experts and health care providers who had actively participated in the establishment of the MWHs to have relatively accurate estimates. Second, we used a macro costing approach which might give different results from other approaches such as micro costing. Despite these limitations, the methods used in this study could be applicable in different settings and we believe the findings are relatively reliable.

## Conclusion

Most essential clinical services and basic amenities were provided at the MWHs. There were differences in the typology of the MWH´s facility. Construction costs of MWHs took the higher portion of the expense of establishing the MWH. Once the construction of a MWH was completed, the cost of operating the MWH was much lower. This study provides essential information that could be used for cost effectiveness analysis of MWHs in the future.

### What is known about this topic

- MWH is considered as a good strategy to overcome inaccessibility of childbirth center due to various reasons;
- Utilization of MWH is increasing in developing countries.

### What this study adds

- The majority of the cost of the MWH was attributed to building construction costs;
- The cost of establishment and running MWH depend on the typologies;
- Estimated unit cost of MWH utilization and delivery was around $17 USD per woman.
